# The urinary microbiome associated with bladder cancer

**DOI:** 10.1038/s41598-018-29054-w

**Published:** 2018-08-14

**Authors:** Viljemka Bučević Popović, Marijan Šitum, Cheryl-Emiliane T. Chow, Luisa S. Chan, Blanka Roje, Janoš Terzić

**Affiliations:** 1 University of Split, Faculty of Science, Department of Chemistry, Split, Croatia; 2 University Hospital Split, Department of Urology, Split, Croatia; 3 Second Genome, Inc., San Francisco, California USA; 4 University of Split, School of Medicine, Department of Immunology, Split, Croatia

## Abstract

Recent findings suggest that human microbiome can influence the development of cancer, but the role of microorganisms in bladder cancer pathogenesis has not been explored yet. The aim of this study was to characterize and compare the urinary microbiome of bladder cancer patients with those of healthy controls. Bacterial communities present in urine specimens collected from 12 male patients diagnosed with bladder cancer, and from 11 healthy, age-matched individuals were analysed using 16S sequencing. Our results show that the most abundant phylum in both groups was *Firmicutes*, followed by *Actinobacteria*, *Bacteroidetes* and *Proteobacteria*. While microbial diversity and overall microbiome composition were not significantly different between groups, we could identify operational taxonomic units (OTUs) that were more abundant in either group. Among those that were significantly enriched in the bladder cancer group, we identified an OTU belonging to genus *Fusobacterium*, a possible protumorigenic pathogen. In an independent sample of 42 bladder cancer tissues, 11 had *Fusobacterium nucleatum* sequences detected by PCR. Three OTUs from genera *Veillonella*, *Streptococcus* and *Corynebacterium* were more abundant in healthy urines. However, due to the limited number of participants additional studies are needed to determine if urinary microbiome is associated with bladder cancer.

## Introduction

Bladder cancer is the ninth most frequent malignant disease, with more than 160,000 deaths per year reported globally. The risk of developing this disease increases with age, and it is diagnosed three times more often in men than in women. Because the majority of new cases are found in people above 65 years of age due to increased life expectancy, it is anticipated that the number of affected individuals will surge in the future^1^.

Apart from environmental and genetic risk factors, researchers have become increasingly aware that microbes inhabiting the human body play an important role for maintenance of health and the development of disease. Microbiome studies, fuelled by the availability of high-throughput DNA-based techniques, have shown that perturbation in the microbiome is associated with a number of human diseases. The vast majority of these studies were performed on the gut, the body niche where most of commensal microorganisms reside, and associations were found between microbiome and diseases such as inflammatory bowel disease, multiple sclerosis, type 1 and 2 diabetes, allergies, asthma, autism, as well as cancer^2^.

The link between cancer and specific microbial agents is well known and it is estimated that microorganisms contribute to up to 20% of human malignancies^3^. The most prominent examples are *Helicobacter pylori* implicated in the development of gastric cancer, and high-risk types of human papillomavirus in cervical cancer^4^. The interaction of microorganisms and their hosts is extremely complex, and a multitude of molecular mechanisms may be envisioned by which they influence oncogenesis, tumour progression and response to anticancer therapy^3,5–7^. Bacteria can directly damage host DNA via genotoxins, such as colibactin produced by some *E. coli* strains, or indirectly by generating reactive oxidative species. Some pathogenic microorganisms manipulate host signalling pathways, exemplified by Wnt/β-catenin pathway which is altered to support cell proliferation in many types of cancers. The microbiome of gastrointestinal tract can also induce chronic inflammation providing a background for tumour development or elicit immunosuppressive responses that may subvert cancer immunosurvellience^3,5^. Finally, bacterial metabolism of host derived metabolites, food components or xenobiotics may result in harmful compounds that may promote tumorigenesis even at distant body sites^3,5^.

Traditionally, bladder epithelium and urine have been considered sterile in healthy individuals. This assumption was based primarily on microbiological urine cultures, best suited for detecting aerobic, fast-growing uropathogens. Evidence has accumulated during the last five years, that the urinary tract also harbours distinct commensal microorganisms^8^. The urinary microbiome reported for healthy people varies considerably due to use of different analytical and urine collection methods. Female urinary microbiome is much better characterized than male. Generally, it may be concluded that there are sex- and age-related differences as well as significant inter-individual variability in urinary microbiome composition^9,10^. Studies have so far explored the changes in urinary microbiome in states such as type 2 diabetes mellitus^11^, overactive bladder^12,13^, urinary incontinence^14–16^, interstitial cystitis^17^, neuropathic bladder^18,19^, sexually transmitted infections^20^ or chronic prostatitis/chronic pelvic pain syndrome^21,22^. The urinary microbiome in urothelial bladder cancer has not been investigated, apart from the pilot study by Xu *et al*.^23^ that reported enrichment of *Streptococcus* sp. in some of the cancer patients. Our study characterized the urinary microbiome of bladder cancer patients and compared it with that of healthy controls to gain insight into the microbiome’s possible role in bladder cancer.

## Results

### Participant characteristics and sequencing data summary

Urine samples were collected from a total of 36 subjects. However, 12 samples failed to provide sufficient DNA for sequencing and one sample did not meet sequencing quality criteria due to low sequencing depth. Supplementary Table S1 displays characteristics of 23 subjects (12 bladder cancer patients and 11 healthy controls) analysed in this study.

Sequencing of urine samples plus extraction control resulted in a total of 22,341,934 raw sequences. These were merged into 9,977,955 paired sequences, with an average read length of 252 base pairs. Filtering for sequence quality and OTU prevalence (min. 10% of samples) reduced the number of sequences to 9,713,510, assigned to 348 OTUs. One of the bladder cancer samples with less than 50,000 reads cut-off (3,722 reads) was excluded from further analysis. Rarefaction curves show that the remaining 23 samples were sequenced to a sufficient depth such that a complete microbiome profile was likely captured for most samples (Supplementary Fig. S1). Classification to the genus level was possible for 95% of sequence reads. A total of 10 bacterial phyla, 19 classes, 26 orders, 61 families and 107 genera were identified. Only two OTUs were detected in the extraction control, one belonged to the *Bacteroides* genus and the other was a chloroplast from the *Streptophyta* order (Supplementary Table S2).

### Microbiome diversity and composition of bladder cancer and healthy urine samples

**Figure 1:**
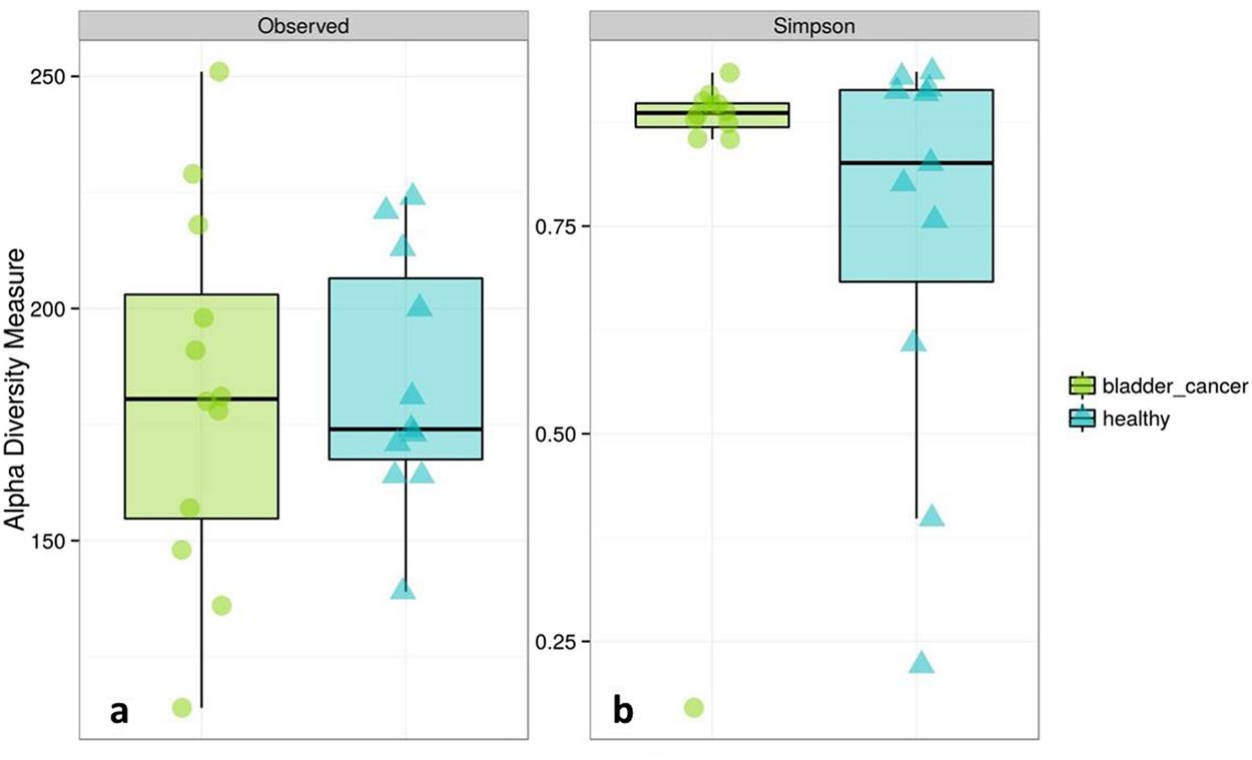
Microbial alpha diversity of urine samples. (**a**) Observed number of OTUs, (**b**) Simpson Index. Both alpha diversity metrics were not statistically different between cancer and healthy samples.

**Figure 2:**
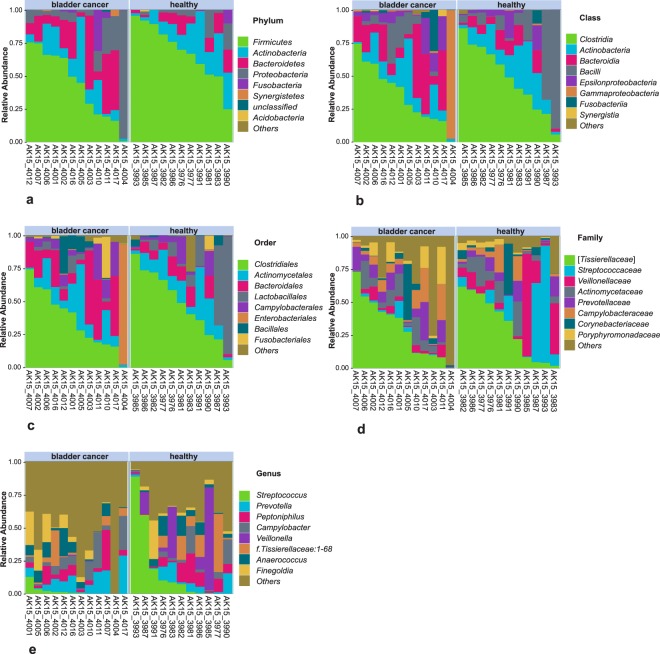
Urinary microbiota of male bladder cancer patients and healthy controls. Most abundant taxa are shown at phylum (**a**), class (**b**), order (**c**), family (**d**) and genus (**e**) level.

Both metrics used to assess differences in microbial alpha diversity (species richness and Simpson index) were not statistically significantly different between cancerous and healthy samples (Fig. 1) possibly due to the low statistical power and/or the variation within the groups. The average number of observed OTUs found within a sample was 182 for the bladder cancer group and 184 for healthy controls (Fig. 1a). The urinary microbiome of bladder cancer patients and healthy controls is shown in Fig. 2. The most abundant phyla included *Firmicutes*, *Actinobacteria*, *Bacteroidetes* and *Proteobacteria*. The most frequently detected genera were *Streptococcus*, *Prevotella*, *Peptoniphilus*, *Campylobacter*, *Veillonella*, *Anaerococcus*, *Finegoldia*, and genus *1–68*, belonging to the *Tissierellaceae* family. A complete list of genera detected in urine samples is given in Supplementary Table S3. A prominent feature of urinary microbiome evidenced from these results is that there is a high degree of inter-individual variability in community composition among study participants in both bladder cancer and healthy subgroups. The microbial composition of the sample collected from one of the bladder cancer patients (AK15_4004 in Fig. 2) was inconsistent with other urine samples; it was dominated by the family *Enterobacteriaceae* with relative abundance of 91% and it was excluded from community structure analysis.

**Figure 3:**
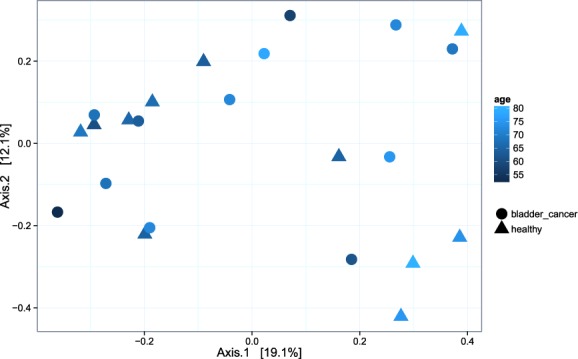
Microbial beta diversity. Dimensional reduction of the Bray-Curtis distance between microbiome samples, using PCoA ordination method, for bladder cancer urines and healthy controls. Data points are coloured according to age in years. Samples do not cluster according to their cancer/healthy status while a moderate clustering according to age is observed.

A PERMANOVA analysis was performed to determine if there is a significant association between microbiome composition and other tested variables such as malignancy, cancer type or patient age. Among those, variations in the urine microbiome were significantly associated only with age across all samples (p = 0.008). A Spearman’s correlation test was used to examine if any of the OTUs were correlated with age, however none of the individual OTUs had adjusted p-values less than 0.05 (Supplementary Fig. S2). This indicates that there is a combination of OTU abundance shifts that contribute to age-related variations in the urine. Bladder cancer urine samples did not cluster in the beta-diversity PCoA, but a moderate clustering according to patients’ age was observed (Fig. 3).

### Community structure reveals differently abundant OTUs in bladder cancer and healthy urine

**Figure 4:**
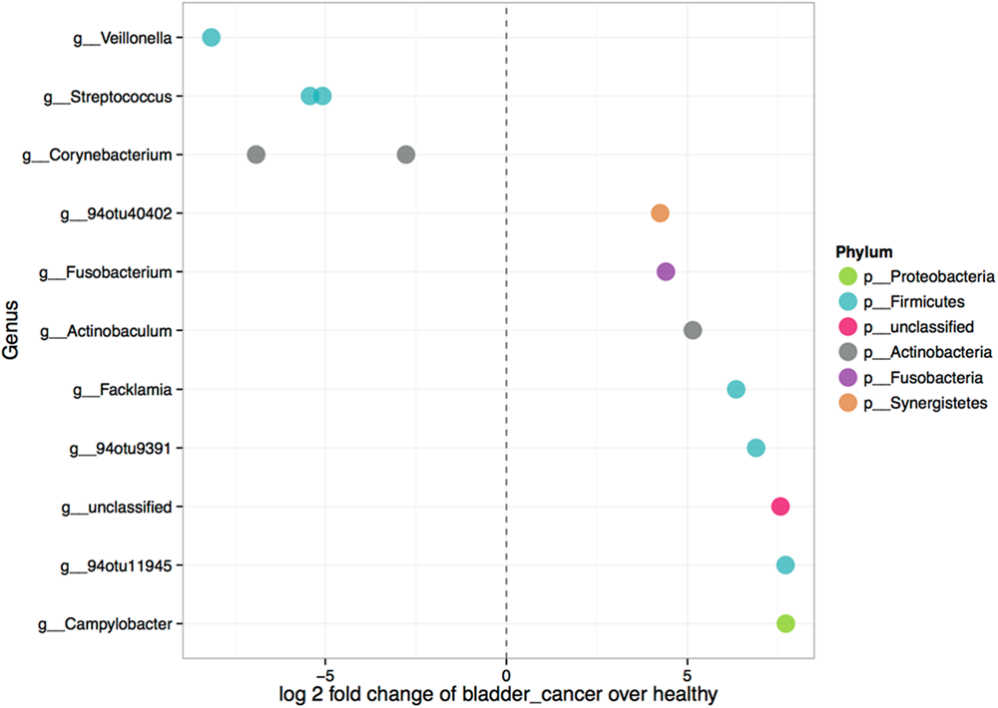
Differently abundant features between urine samples from bladder cancer patients and healthy controls. Each point represents an OTU belonging to respective genus. 94otu4042 was identified as *Jonquetella anthropi*, while 94otu9391 and 94otu11945 belong to family *Ruminococcaceae* and were identified by searching the SILVA rRNA database as *Ruminococcaceae UCG-002* and *Subdoligranulum*, respectively. Features were considered significant if their false discovery rate-corrected p-value was less than or equal to 0.05, and the absolute value of the log2 fold change was greater than or equal to 1.

While there was no significant difference in microbiome composition in terms of overall diversity or composition at the phylum or family level, specific OTUs were identified that exhibited significant differences (p < 0.05) in abundance between cancer and healthy samples (Fig. 4). Eight OTUs were enriched in urine of bladder cancer patients including the *Fusobacterium, Actinobaculum*, *Facklamia* and *Campylobacter* genera, and two OTUs belonging to *Ruminococcaceae* family that were further identified as *Subdoligranulum* (94otu11945) and *Ruminococcaceae UCG-002* (94otu9391) by comparing them against the SILVA rRNA database^24^. Three OTUs identified at strain level were *Campylobacter hominis*, *Actinobaculum massiliense*, and *Jonquetella anthropi* (94otu40402). Five OTUs were enriched in healthy samples from the genera *Veillonella*, *Streptococcus*, *Corynebacterium*; these OTUs were further identified as *Veillonella dispar* at species, and *Streptococcus cristatus*, *Corynebacterium appendicis* and *Corynebacterium sp*. at strain level.

### Analysis of bladder cancer tissue samples by *F. nucleatum* specific PCR

In order to determine the frequency of cancerous tissue that is positive for *Fusobacterium nucleatum*, a possible protumorigenic pathogen, we have tested an independent set of urinary bladder cancer tissues from our biobank by *F. nucleatum* specific PCR reaction (Supplementary Fig. S3). We tested tumour tissue from 42 patients and 11 of them (26%) were positive for *Fusobacterium nucleatum*.

## Discussion

In this study, we have characterized urinary microbial communities of male patients diagnosed with primary or recurrent bladder cancer and compared it with those of disease free, age-matched controls. Although we did not observe major differences in overall microbiome profiles, we identified several OTUs that were significantly over-represented in bladder cancer or healthy subgroup. These differences suggest a possible role for urinary microbiome in bladder cancer pathogenesis that merits further evaluation.

A reduction in microbial diversity in urine of bladder cancer patients was not detected in our study (Fig. 1). While reduced diversity in the gut has been commonly linked with the state of disease^25^, changes in microbiome diversity have not been consistently associated with urinary tract disorders. Reduced bacterial diversity was observed in interstitial cystitis^17^, increased diversity was found in urgency urinary incontinence^15^ and chronic prostatitis^21^, while no changes in microbial diversity could be associated with overactive^12^ or neuropathic bladder symptomatology^18^. It may be that the abundance of specific bacteria in urine is more important than the total number of bacterial taxa present. Future studies could potentially address this question by investigating the total microbial biomass or abundance present in the urine alongside investigations into the diversity and composition of the microbiome.

Inter-individual variability, observed between microbiomes of the participants in our study, has been repeatedly demonstrated in publications on urinary microbiome^10,11,19,20^, which makes it challenging to define what constitutes a ‘core’ bladder microbiome. However, the most abundant genera present in our samples, with the sole exception of *Tissierellaceae:1–68* (shown in Fig. 2e), were repeatedly found in urine of male individuals^10,19,20,26^ and may be considered typical urinary bacteria. There are additional similarities in urinary microbiome composition when considering a complete list of genera observed in our samples and results obtained by others (Supplementary Table S3).

Our comparison of bladder cancer versus healthy urine samples revealed bacterial taxa that were overrepresented in one of the sample subgroups (Fig. 4). Of note, among those OTUs that were more abundant in bladder cancer group, an OTU belonging to genus *Fusobacterium* was detected. This was due to the increased relative abundance of *Fusobacterium* in four of the bladder cancer samples, compared to only one healthy control sample (Supplementary Fig. S4). *Fusobacterium* is a common constituent of the oral microbiome, but also an opportunistic pathogen with recognized carcinogenic potential^27,28^. It has been found associated with colorectal cancer in several studies^29–32^. Fusobacterial DNA was also detected in pancreatic^33^, breast^34^, esophageal^35^ and laryngeal^36^ cancer tissue, but this is to our knowledge, the first report on possible association of *Fusobacterium* with urothelial carcinomas, at least in some bladder cancer patients. A proposed mechanism by which *Fusobacterium nucleatum* drives tumorigenesis involves activation of β-catenin cell signalling pathway leading to cell proliferation^37,38^. A study by Kostić *et al*.^39^, based on human sample analysis and mice model, suggests that, through recruitment of tumour-infiltrating immune cells, *Fusobacteria* generate a proinflammatory environment which supports tumour progression. There is also evidence that *F. nucleatum* inhibits NK cell cytotoxicity and T cell activity, thereby promoting immune evasion, which is one of the hallmarks of cancer^40^.

Enrichment of *F. nucleatum* in cancerous tissue seems to be mediated by binding of fusobacterial Fap2 lectin to tumour-displayed D-galactose-β(1-3)-N-acetyl-D-galactosamine (Gal-GalN Ac)^41^. A recently published pilot study demonstrated that in addition to colorectal carcinomas, various tumour types display Gal-GalNAc^42^. Although only moderate levels of Gal-GalNAc were found in urothelial carcinomas, the microbiome analysis undertaken in our study suggests that bladder cancer tissue can also be colonized by *F. nucleatum*. To further test this, we have investigated the presence of *F. nucelatum* in a collection of bladder cancer tissue samples. The PCR-based analysis showed that *F. nucleatum* is indeed present in approximately one quarter of the tested samples.

The most prominent OTU enriched in bladder cancer urines in our study (Fig. 4) was identified as *Campylobacter hominis*. Studies have shown that *Campylobacter* species are potentially pathogenic as they are able to produce toxins, invade epithelial cells, and avoid host immune responses. Similarly, *Campylobacter* species were found over-represented together with *Fusobacterium* in colorectal cancer^43^ and esophageal biopsies^44^.

Amongst other OTUs that were significantly more abundant in cancer patients, an OTU belonging to genera *Jonquetella* was detected. *Jonquetella* presence is potentially characteristic of urine microbiomes from individuals aged 70 and older^10^. Given the relatively small sample size, it is interesting that we could observe clustering of samples according to age (Fig. 3), even though the study cohort already consisted of older individuals (Supplementary Table S1). These results support previous conclusion by Lewis *et al*. that aging modifies the composition of microbial communities in both the bladder and the gut^10^, although none of the individual OTUs detected in this study passed the false discovery rate adjustment (Supplementary Fig. S2). Whether these shifts in microbiome composition that occur with aging increase cancer risks remains to be investigated.

Apart from being oncogenic, commensal microbiota may also provide beneficial, tumour-suppressive effects to the human host^7^. The concept that specific bacteria could protect against development of a malignant disease is particularly straightforward when considering urinary bladder cancer, because this is the only malignancy treated by a live microorganism, *Mycobacterium bovis* bacille Calmette-Guérin (BCG). Despite being used for more than 40 years, the molecular details of its therapeutic action are not fully elucidated. A proposed model suggests that BCG attaches to urothelial cells, which is followed by BCG internalization by bladder cancer cells and initiation of immune responses that destroy cancerous tissue^45^.

It could be envisioned that similarly to BCG, certain commensal bacteria, residing naturally in the healthy bladder, could serve the function of tumour surveillance or act beneficially in a different manner. In this study, five OTUs were found to be increased in healthy bladder and they included *Streptococcus*, *Veillonella*, and *Corynebacterium* species. *Corynebacterium* might be a typical urine component in healthy men as opposed to *Lactobacillus* which is prevalent in women^19^. *Streptococcus*, and to a lesser extent *Veillonella*, have repeatedly been observed in urine of healthy men^9,19,26^. In addition, characterization of microbial populations in specimens of another urological malignancy, prostate cancer, also showed the statistically significant enrichment of *Streptococcus* in nontumorous tissue^46^. However, it must be noted the opposite was observed in the urinary microbiome study by Xu *et al*.^23^, where *Streptococcus* abundance was elevated in 5 of the 8 urothelial carcinoma patients compared to near zero abundance in 5 out of 6 healthy individuals. Therefore, additional studies are needed to clarify the association of *Streptococcus* abundance with either cancer or healthy status.

Our study on bladder cancer microbiome had some limitations. We used clean-catch midstream urine to sample bladder microbiome, which is from a patient point a far less invasive method then suprapubic aspiration or catheterization. We cannot exclude the possibility of contamination with bacteria colonizing urethra. On the other hand, microorganisms that adhere to urothelial cells or form biofilms in the bladder wall may be underrepresented in urine samples. However, screening of bladder cancer tissue samples for *F. nucleatum* by specific PCR reaction has shown that this microorganism is indeed present in cancerous bladder tissue. This suggests that voided midstream urine collection can be used to investigate urinary bladder microbiome despite its obvious limitations.

This study included only male patients. Men are at considerably higher risk of developing bladder cancer, which may be explained by the effects of sex hormones or gender differences in metabolic detoxification of carcinogens^47,48^. It would be interesting to explore if sex-related variations in urinary microbiome composition also contribute to different bladder cancer risks. For example, *Lactobacillus*, a typical member of the female urinary microbiome, has been found to induce tumour regression in a murine model of bladder cancer^49^.

As with other studies comparing disease versus healthy microbiome, it is not possible to say whether the microbial alterations are the cause or the consequence of the disease. Further longitudinal studies with a larger sample number at different stages of tumorigenesis and animal model studies will be needed to clarify the role of microbiome in bladder cancer formation and progression.

The main strength of the study is the novel insight on subtle changes of urinary microbiome in bladder cancer, as evidenced by increased or decreased abundances for a number of OTUs. Additionally, these results are important because the male urinary microbiome is often overlooked in urological studies, as the field is much more focused on female urogenital pathologies. More studies like this are needed to further define the core microbiome in both sexes and evaluate how it changes in specific disease states.

In conclusion, the 16S rDNA gene sequencing-based approach used in this work enabled us to characterize urinary bladder microbiome and detect differences in the relative abundance of specific bacteria in bladder cancer patients, with *Fusobacteria* as a possibly important representative. Whether observed differences contribute to bladder cancer development remains to be elucidated. A better understanding of the role of microbiome in bladder cancer could direct urologists to novel diagnostic and prognostic options, as well as to more personalized treatments and microbiome-targeted therapeutic interventions.

## Methods

### Subject recruitment and sample collection

The study began following approval from the Ethics Committee of the University Hospital in Split. Thirty six Caucasian men were recruited at the Department of Urology, University Hospital Split, between October 2015 and October 2016.

The bladder cancer group contained 17 males diagnosed with primary or recurrent non muscle-invasive tumours (TNM grade Ta, T1 or CIS). Initial suspicion of urinary bladder cancer was reached after a physical and ultrasound examination by a trained urologist. The diagnosis was subsequently confirmed by cystoscopy and tumour tissue analysis by certified pathologist, followed by cancer tissue removal by trans-urethral resection (TUR) approach. Urine samples for microbiome analysis were collected after ultrasound examination and prior to cystoscopy. Patients with recurrent tumours were previously surgically treated but because of the mild nature of their tumours (low grade, Ta stage) none of the other therapy modalities were needed (treatment with Bacillus Calmette-Guérin (BCG), intravesical chemotherapy or radiotherapy). Initial surgery among patients with recurrent tumours was done more than one year before urine sample collection.

The control group had 19 healthy individuals who visited a urologist for prostate cancer screening check-up. None of the healthy controls had prostate cancer or indications for prostate biopsy or cystoscopy.

Only individuals (patients and healthy controls), that were not taking antibiotics for any reason (urinary or non-urinary) for one month prior to urine collection, were included into our study. Exclusion criteria for both groups were positive history of sexually transmitted or recent urinary infections, diabetes and obesity. Additional participant characteristics are given in Supplementary Table S1. All experiments were performed in accordance with relevant guidelines and regulations and participants gave written informed consent for urine collection and analysis for research purposes. Clean catch, midstream urine was collected from all participants and stored at −80 °C until DNA extraction.

### DNA isolation from urine

Urine specimens (30 ml) were thawed and centrifuged at 7500 g, 4 °C for 10 minutes. The pellet was used for DNA extraction using PowerSoil^@^DNA Isolation Kit (MoBio Laboratories, Inc.), performed according to manufacturer’s protocol. To avoid environmental contamination, all isolations from urine samples and from the reagent-only extraction control were carried out within a PCR hood. Isolated DNA samples were placed at −20 °C until PCR amplification. DNA was quantified via the Qubit^@^ Quant-iT dsDNA High Sensitivity Kit 7 (Invitrogen, Life Technologies).

### 16S rRNA gene library preparation and MiSeq sequencing

PCR amplification of 16S rDNA, sequencing and analyses were performed by Second Genome, Inc. 16S rRNA gene V4 region was amplified with 515F-806R fusion primers that incorporate Illumina adapters and indexing barcodes^50^. PCR products were quantified using Quant-iT™ PicoGreen™ dsDNA Assay Kit from Invitrogen (Life Technologies, Grand Island, NY), pooled in equal molar ratios, and sequenced for 2 × 250 cycles on the Illumina MiSeq platform (Illumina, San Diego, CA).

### Bioinformatics and statistical analyses

Sequenced paired-end reads were processed using USEARCH^51^. All sequences hitting a unique strain in an in-house strains database with an identity ≥99% were assigned a strain Operational Taxonomic Unit (OTU). The remaining non-strain sequences were quality filtered, dereplicated and then clustered at 97% by UPARSE^52^. Representative OTU sequences were assigned a taxonomic classification at 80% confidence cut-off via mothur’s bayesian classifier^53^, against the Greengenes reference database of 16S rRNA gene sequences^54^ clustered at 99% OTUs. A prevalence filter was used to remove spurious OTUs that were observed in less than 10% of the sample set.

Diversity within samples (alpha diversity) was evaluated as richness and Simpson diversity. Richness is the number of observed unique Operational Taxonomic Units (OTUs), and Simpson Index considers both the richness and the abundance of each OTU. Dissimilarity between samples (beta diversity) was assessed using the Bray-Curtis dissimilarity measure^55^. To visualize inter-sample relationships, Principal Coordinates Analysis (PCoA) was performed.

Differences in the overall microbial composition between bladder cancer and healthy samples were assessed by permutational analysis of variance, PERMANOVA^56^. To identify taxa that were significantly different between bladder cancer and healthy samples, we used DESeq2 package^57^, described for microbiome applications^58^. DESeq2 was run under default settings and q-values were calculated with the Benjamini-Hochberg procedure to correct p-values and control for false discovery rates.

### PCR analysis of bladder tissue samples

*Fusobacterium nucleatum* specific PCR reaction was performed with previously collected urinary bladder cancer tissue samples from our biobank. Patient data, details on DNA isolation and PCR protocol are given in Supplementary Methods.

### Data availability

The datasets generated during the current study are available in the European Nucleotide Archive, accession number: PRJEB22327.

## Electronic supplementary material

Supplementary Information
