# Supplementary material for: The urinary microbiome associated with bladder cancer
Source: Sci Rep. 2018 Aug 14;8:12157. doi: 10.1038/s41598-018-29054-w (PMC6092344; doi:10.1038/s41598-018-29054-w)
Supplement: Supplementary file 1 — Supplementary Information [file 41598_2018_29054_MOESM1_ESM.pdf]

The urinary microbiome associated with bladder cancer

Viljemka Bučević Popović[a], Marijan Šitum[b], Cheryl-Emiliane T. Chow[c], Luisa S. Chan[c], Blanka Roje[d], Janoš Terzić[d,*]

[a]University of Split, Faculty of Science, Department of Chemistry, Split, Croatia

[b]University Hospital Split, Department of Urology, Split, Croatia

[c]Second Genome Inc., San Francisco, California, USA

[d]University of Split, School of Medicine, Department of Immunology, Split, Croatia

Supplementary Methods

Bladder cancer tissue collection

Bladder cancer tissues were collected from 42 patients with primary urinary bladder cancer surgically treated at the University Hospital in Split, from June 2009 to October 2010. All patients gave informed consent and the study was approved by the Ethics Committee of the University Hospital Split. Thirty-five patients were male and 7 were female with the mean age of 69.9±13.5. Patients underwent transurethral bladder resection (TUR) during which tumour samples were collected and immediately frozen in liquid nitrogen. Tumour specimens from all patients were paraffin embedded and evaluated by an experienced pathologist according to the TNM classification. Of the 42 samples, 31 had Ta-stage and 11 had T1-stage urothelial cancer.

DNA isolation from bladder tissue samples and PCR protocol

DNA from tumour tissues was isolated using TRIZOL reagent. The amount of approximately 200 ng of DNA was used in each PCR reaction. The following primer set was used for *Fusobacterium nucleatum* detection: foreward primer, 5'-GGATTTATTGGGCGTAAAGC-3'; reverse primer, 5'-GGCATTCCTACAAATATCTACGAA-3'. PCR reaction was performed as follows: initial denaturation step at 95 °C for 10 min, followed by 35 cycles of 1 minute at 95 °C, 1 minute at 51 °C and 1 minute at 72 °C, with final extension step at 72 °C for 5 min. Negative control was PCR reaction mixture without any DNA, while positive control contained *F. nucleatum* DNA that was confirmed by sequencing.

Supplementary Tables and Figures

Table S1. Participant characteristics. Urine specimens were collected from a total of 36 individuals. Details are given only for the proportion of bladder cancer patients with urines that provided enough DNA for successful sequencing analysis of microbiomes.

	**Bladder cancer**	**Healthy**
**Number of participants in the study**	17	19
**Median age (years)**	69 (54-82)	70 (61-82)
**Number of participants with completed microbiome profiles**	12	11
**Median age (years)**	67 (54-82)	67 (61-80)
**Disease state**		N/A
Primary tumor	7	
Recurrent tumor	5	
**Malignancy grade**		N/A
High	4	
Low	8	
**TNM Stage**		N/A
Ta	10	
Ta + Cis	1	
T1	1	

Table S2. OTUs identified in the negative extraction control. Two OTUs were observed in 178 reads sequenced from the negative extraction control. Annotations to genus level and relative abundances are shown.

Abundance	Taxonomic annotation
0.579	"o__Bacteroidales","f__ Bacteroidaceae ","g__97otu98108"
0.421	"o__Streptophyta","f__unclassified","g__unclassified"

Table S3. A list of genera identified in urine samples. Bacterial genera detected in urine of male individuals in previous studies[10,19,20,25] are highlighted in bold.

"p__Acidobacteria","c__[Chloracidobacteria]","o__RB41","f__Ellin6075","g__unclassified"
**"p__Actinobacteria","c__Actinobacteria","o__Actinomycetales","f__Actinomycetaceae","g__Actinobaculum"**
**"p__Actinobacteria","c__Actinobacteria","o__Actinomycetales","f__Actinomycetaceae","g__Actinomyces"**
"p__Actinobacteria","c__Actinobacteria","o__Actinomycetales","f__Actinomycetaceae","g__Arcanobacterium"
**"p__Actinobacteria","c__Actinobacteria","o__Actinomycetales","f__Actinomycetaceae","g__Mobiluncus"**
**"p__Actinobacteria","c__Actinobacteria","o__Actinomycetales","f__Actinomycetaceae","g__Varibaculum"**
"p__Actinobacteria","c__Actinobacteria","o__Actinomycetales","f__Brevibacteriaceae","g__Brevibacterium"
**"p__Actinobacteria","c__Actinobacteria","o__Actinomycetales","f__Corynebacteriaceae","g__Corynebacterium"**
"p__Actinobacteria","c__Actinobacteria","o__Actinomycetales","f__Dermabacteraceae","g__Dermabacter"
"p__Actinobacteria","c__Actinobacteria","o__Actinomycetales","f__Intrasporangiaceae","g__unclassified"
"p__Actinobacteria","c__Actinobacteria","o__Actinomycetales","f__Microbacteriaceae","g__Pseudoclavibacter"
**"p__Actinobacteria","c__Actinobacteria","o__Actinomycetales","f__Micrococcaceae","g__Kocuria"**
"p__Actinobacteria","c__Actinobacteria","o__Actinomycetales","f__Micrococcaceae","g__Micrococcus"
**"p__Actinobacteria","c__Actinobacteria","o__Actinomycetales","f__Micrococcaceae","g__Rothia"**
"p__Actinobacteria","c__Actinobacteria","o__Actinomycetales","f__Micrococcaceae","g__unclassified"
"p__Actinobacteria","c__Actinobacteria","o__Actinomycetales","f__Propionibacteriaceae","g__94otu20159"
"p__Actinobacteria","c__Actinobacteria","o__Actinomycetales","f__Propionibacteriaceae","g__94otu39580"
"p__Actinobacteria","c__Actinobacteria","o__Actinomycetales","f__Pseudonocardiaceae","g__unclassified"
"p__Actinobacteria","c__Actinobacteria","o__Bifidobacteriales","f__Bifidobacteriaceae","g__Alloscardovia"
"p__Actinobacteria","c__Actinobacteria","o__Bifidobacteriales","f__Bifidobacteriaceae","g__Bifidobacterium"
"p__Actinobacteria","c__Coriobacteriia","o__Coriobacteriales","f__Coriobacteriaceae","g__94otu40707"
"p__Actinobacteria","c__Coriobacteriia","o__Coriobacteriales","f__Coriobacteriaceae","g__94otu8264"
**"p__Actinobacteria","c__Coriobacteriia","o__Coriobacteriales","f__Coriobacteriaceae","g__Atopobium"**
"p__Actinobacteria","c__Coriobacteriia","o__Coriobacteriales","f__Coriobacteriaceae","g__Collinsella"
"p__Actinobacteria","c__unclassified","o__unclassified","f__unclassified","g__unclassified"
**"p__Bacteroidetes","c__Bacteroidia","o__Bacteroidales","f__[Paraprevotellaceae]","g__[Prevotella]"**
"p__Bacteroidetes","c__Bacteroidia","o__Bacteroidales","f__Bacteroidaceae","g__Bacteroides"
"p__Bacteroidetes","c__Bacteroidia","o__Bacteroidales","f__Porphyromonadaceae","g__Parabacteroides"
**"p__Bacteroidetes","c__Bacteroidia","o__Bacteroidales","f__Porphyromonadaceae","g__Porphyromonas"**
**"p__Bacteroidetes","c__Bacteroidia","o__Bacteroidales","f__Prevotellaceae","g__Prevotella"**
"p__Bacteroidetes","c__Bacteroidia","o__Bacteroidales","f__unclassified","g__unclassified"
"p__Bacteroidetes","c__Flavobacteriia","o__Flavobacteriales","f__[Weeksellaceae]","g__Chryseobacterium"
"p__Bacteroidetes","c__unclassified","o__unclassified","f__unclassified","g__unclassified"
"p__Cyanobacteria","c__Chloroplast","o__Streptophyta","f__91otu7296","g__94otu15236"
"p__Cyanobacteria","c__Chloroplast","o__Streptophyta","f__unclassified","g__unclassified"
"p__Firmicutes","c__Bacilli","o__Bacillales","f__Staphylococcaceae","g__Jeotgalicoccus"
**"p__Firmicutes","c__Bacilli","o__Bacillales","f__Staphylococcaceae","g__Staphylococcus"**
"p__Firmicutes","c__Bacilli","o__Gemellales","f__Gemellaceae","g__94otu3263"
**"p__Firmicutes","c__Bacilli","o__Lactobacillales","f__Aerococcaceae","g__Aerococcus"**
**"p__Firmicutes","c__Bacilli","o__Lactobacillales","f__Aerococcaceae","g__Facklamia"**

(Continued)

**"p__Firmicutes","c__Bacilli","o__Lactobacillales","f__Carnobacteriaceae","g__Granulicatella"**
**"p__Firmicutes","c__Bacilli","o__Lactobacillales","f__Enterococcaceae","g__Enterococcus"**
**"p__Firmicutes","c__Bacilli","o__Lactobacillales","f__Lactobacillaceae","g__Lactobacillus"**
**"p__Firmicutes","c__Bacilli","o__Lactobacillales","f__Streptococcaceae","g__Streptococcus"**
"p__Firmicutes","c__Bacilli","o__Lactobacillales","f__unclassified","g__unclassified"
"p__Firmicutes","c__Clostridia","o__Clostridiales","f__[Mogibacteriaceae]","g__94otu769"
"p__Firmicutes","c__Clostridia","o__Clostridiales","f__[Mogibacteriaceae]","g__Mogibacterium"
"p__Firmicutes","c__Clostridia","o__Clostridiales","f__[Tissierellaceae]","g__1-68"
"p__Firmicutes","c__Clostridia","o__Clostridiales","f__[Tissierellaceae]","g__94otu32621"
"p__Firmicutes","c__Clostridia","o__Clostridiales","f__[Tissierellaceae]","g__94otu43682"
**"p__Firmicutes","c__Clostridia","o__Clostridiales","f__[Tissierellaceae]","g__Anaerococcus"**
**"p__Firmicutes","c__Clostridia","o__Clostridiales","f__[Tissierellaceae]","g__Finegoldia"**
**"p__Firmicutes","c__Clostridia","o__Clostridiales","f__[Tissierellaceae]","g__Gallicola"**
"p__Firmicutes","c__Clostridia","o__Clostridiales","f__[Tissierellaceae]","g__Helcococcus"
**"p__Firmicutes","c__Clostridia","o__Clostridiales","f__[Tissierellaceae]","g__Peptoniphilus"**
"p__Firmicutes","c__Clostridia","o__Clostridiales","f__[Tissierellaceae]","g__ph2"
"p__Firmicutes","c__Clostridia","o__Clostridiales","f__[Tissierellaceae]","g__WAL_1855D"
"p__Firmicutes","c__Clostridia","o__Clostridiales","f__91otu18736","g__94otu38091"
"p__Firmicutes","c__Clostridia","o__Clostridiales","f__91otu3623","g__94otu37889"
"p__Firmicutes","c__Clostridia","o__Clostridiales","f__91otu452","g__94otu18036"
"p__Firmicutes","c__Clostridia","o__Clostridiales","f__91otu5947","g__94otu42223"
**"p__Firmicutes","c__Clostridia","o__Clostridiales","f__Clostridiaceae","g__Clostridium"**
"p__Firmicutes","c__Clostridia","o__Clostridiales","f__Lachnospiraceae","g__94otu10116"
"p__Firmicutes","c__Clostridia","o__Clostridiales","f__Lachnospiraceae","g__94otu19924"
"p__Firmicutes","c__Clostridia","o__Clostridiales","f__Lachnospiraceae","g__94otu9002"
"p__Firmicutes","c__Clostridia","o__Clostridiales","f__Lachnospiraceae","g__Blautia"
"p__Firmicutes","c__Clostridia","o__Clostridiales","f__Lachnospiraceae","g__Coprococcus"
**"p__Firmicutes","c__Clostridia","o__Clostridiales","f__Lachnospiraceae","g__Dorea"**
"p__Firmicutes","c__Clostridia","o__Clostridiales","f__Lachnospiraceae","g__Lachnospira"
"p__Firmicutes","c__Clostridia","o__Clostridiales","f__Lachnospiraceae","g__Moryella"
"p__Firmicutes","c__Clostridia","o__Clostridiales","f__Lachnospiraceae","g__unclassified"
**"p__Firmicutes","c__Clostridia","o__Clostridiales","f__Peptococcaceae","g__Peptococcus"**
"p__Firmicutes","c__Clostridia","o__Clostridiales","f__Peptostreptococcaceae","g__94otu13105"
"p__Firmicutes","c__Clostridia","o__Clostridiales","f__Peptostreptococcaceae","g__94otu37150"
"p__Firmicutes","c__Clostridia","o__Clostridiales","f__Ruminococcaceae","g__94otu11945"
"p__Firmicutes","c__Clostridia","o__Clostridiales","f__Ruminococcaceae","g__94otu27202"
"p__Firmicutes","c__Clostridia","o__Clostridiales","f__Ruminococcaceae","g__94otu9391"
"p__Firmicutes","c__Clostridia","o__Clostridiales","f__Ruminococcaceae","g__Faecalibacterium"
"p__Firmicutes","c__Clostridia","o__Clostridiales","f__Ruminococcaceae","g__Oscillospira"
"p__Firmicutes","c__Clostridia","o__Clostridiales","f__Ruminococcaceae","g__Ruminococcus"
"p__Firmicutes","c__Clostridia","o__Clostridiales","f__Ruminococcaceae","g__unclassified"
"p__Firmicutes","c__Clostridia","o__Clostridiales","f__unclassified","g__unclassified"
**"p__Firmicutes","c__Clostridia","o__Clostridiales","f__Veillonellaceae","g__Dialister"**
**"p__Firmicutes","c__Clostridia","o__Clostridiales","f__Veillonellaceae","g__Veillonella"**

(Continued)

**"p__Fusobacteria","c__Fusobacteriia","o__Fusobacteriales","f__Fusobacteriaceae","g__Fusobacterium"**
"p__Fusobacteria","c__Fusobacteriia","o__Fusobacteriales","f__Leptotrichiaceae","g__Streptobacillus"
"p__Proteobacteria","c__Alphaproteobacteria","o__Caulobacterales","f__Caulobacteraceae","g__94otu5246"
"p__Proteobacteria","c__Alphaproteobacteria","o__Rhodobacterales","f__Rhodobacteraceae","g__Paracoccus"
"p__Proteobacteria","c__Alphaproteobacteria","o__Sphingomonadales","f__Erythrobacteraceae","g__94otu31357"
**"p__Proteobacteria","c__Alphaproteobacteria","o__Sphingomonadales","f__Sphingomonadaceae","g__Sphingomonas"**
**"p__Proteobacteria","c__Betaproteobacteria","o__Burkholderiales","f__Alcaligenaceae","g__Sutterella"**
"p__Proteobacteria","c__Betaproteobacteria","o__Burkholderiales","f__Comamonadaceae","g__Comamonas"
"p__Proteobacteria","c__Betaproteobacteria","o__Burkholderiales","f__Comamonadaceae","g__unclassified"
"p__Proteobacteria","c__Betaproteobacteria","o__Burkholderiales","f__Oxalobacteraceae","g__unclassified"
"p__Proteobacteria","c__Deltaproteobacteria","o__Desulfovibrionales","f__Desulfovibrionaceae","g__Bilophila"
**"p__Proteobacteria","c__Epsilonproteobacteria","o__Campylobacterales","f__Campylobacteraceae","g__Campylobacter"**
"p__Proteobacteria","c__Gammaproteobacteria","o__Enterobacteriales","f__Enterobacteriaceae","g__unclassified"
"p__Proteobacteria","c__Gammaproteobacteria","o__Pasteurellales","f__Pasteurellaceae","g__Aggregatibacter"
**"p__Proteobacteria","c__Gammaproteobacteria","o__Pasteurellales","f__Pasteurellaceae","g__Haemophilus"**
**"p__Proteobacteria","c__Gammaproteobacteria","o__Pseudomonadales","f__Moraxellaceae","g__Acinetobacter"**
"p__Proteobacteria","c__Gammaproteobacteria","o__Pseudomonadales","f__Moraxellaceae","g__Enhydrobacter"
**"p__Proteobacteria","c__Gammaproteobacteria","o__Pseudomonadales","f__Pseudomonadaceae","g__Pseudomonas"**
"p__Proteobacteria","c__Gammaproteobacteria","o__Pseudomonadales","f__Pseudomonadaceae","g__unclassified"
"p__Spirochaetes","c__Spirochaetes","o__Spirochaetales","f__Spirochaetaceae","g__Treponema"
"p__Synergistetes","c__Synergistia","o__Synergistales","f__Dethiosulfovibrionaceae","g__94otu40402"
"p__Synergistetes","c__Synergistia","o__Synergistales","f__Dethiosulfovibrionaceae","g__Pyramidobacter"

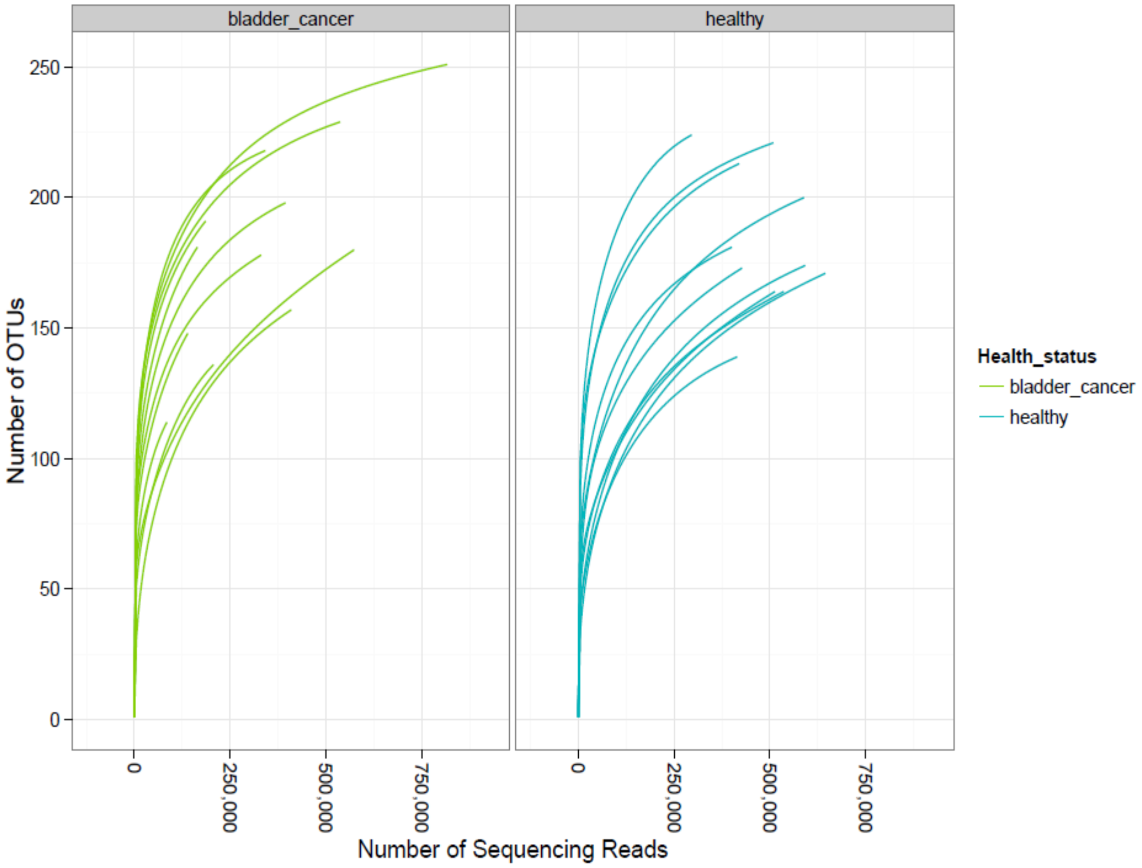

Figure S1. Rarefaction curves. Average number of OTUs detected versus sequencing library size for urine samples collected from bladder cancer patients and healthy controls.

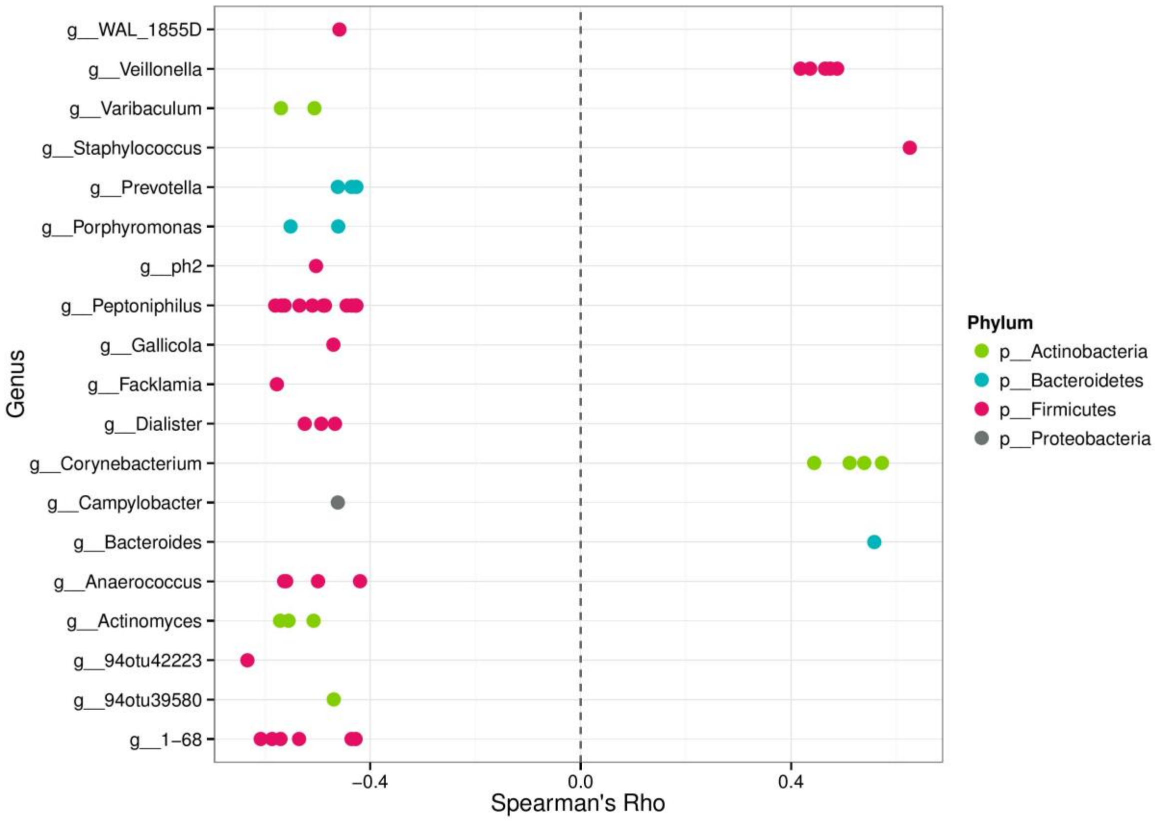

Figure S2. Variations in the urine associated with age. A Spearman's correlation test (Spearman's Rho) was used to investigate OTU level abundance shifts with age. None of the individual OTUs passed false discovery rate adjustment. Those OTUs with unadjusted p-values less than 0.05 are shown.

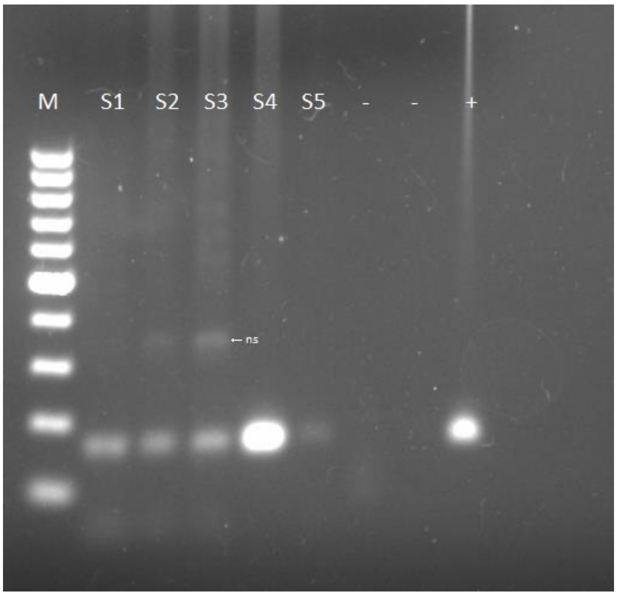

Figure S3. Analysis of bladder cancer tissue samples by *Fusobacterium nucleatum* specific PCR. Result of PCR analysis of 5 different bladder tumour samples are shown (**S1-S5**). The size of the amplicon is 163 pb. **NS** – nonspecific amplification; **M** – 100 bp ladder, **-** – negative control; **+** – positive control

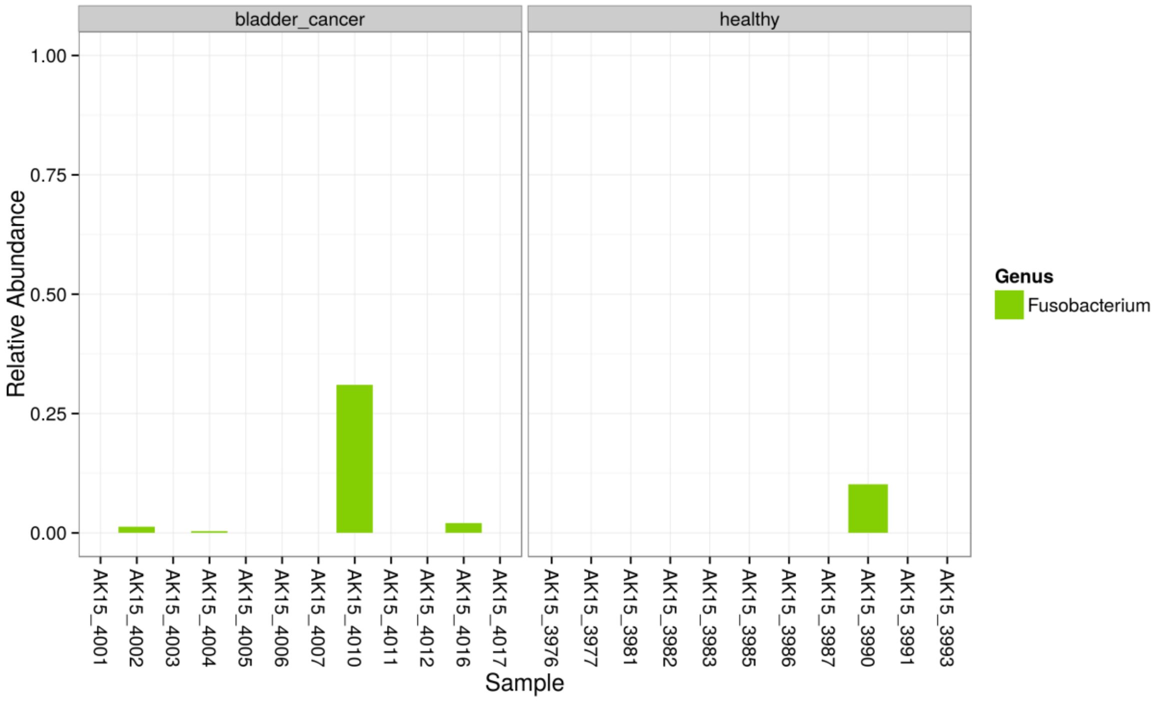

Figure S4. The abundance of *Fusobacteria* in urinary microbiome. The relative abundances of genus *Fusobacterium* in urine of bladder cancer patients and healthy controls are shown.
